# Commentary: Amyotrophic Lateral Sclerosis and Myasthenia Gravis Overlap Syndrome: A Review of Two Cases and the Associated Literature

**DOI:** 10.3389/fneur.2017.00356

**Published:** 2017-07-27

**Authors:** Ian Paul Johnson, Patrizia Longone

**Affiliations:** ^1^Anatomy and Pathology, Adelaide Medical School, University of Adelaide, Adelaide, SA, Australia; ^2^Molecular Neurobiology Unit, Experimental Neurology, Fondazione Santa Lucia, Rome, Italy

**Keywords:** amyotrophic lateral sclerosis, myasthenia overlap syndrome, neuromuscular junction, neurotrophic support, “dying back” hypothesis

The recent paper by Tai et al. (1) is a timely reminder that age-related neurodegenerative diseases rarely exist in isolation, and that we should make every effort to use points of commonality to better understand the pathophysiology of these largely untreatable conditions. In their paper (1), the authors present two cases of amyotrophic lateral sclerosis (ALS) coexisting with myasthenia gravis (MG) and review the literature on this. They report that motoneuronal death in this rare syndrome can be preceded or followed by MG and conclude that the weight of evidence points toward a common neuromuscular defect operating in both diseases. This is important because the interdependence of motoneurones and their peripheral targets is well known. This was shown in the early studies of Hamburger (2, 3) and confirmed in more recent studies of the myotrophic and neuroprotective effect of muscle-derived molecules in experimental motoneuronal death and models of human motoneuronal diseases (4–6). Since MG is characterized by autoantibodies to the acetylcholine receptor at the neuromuscular junction (NMJ), Tai et al. (1) unsurprisingly implicate autoimmune damage at the NMJ in the pathogenesis of ALS. Further studies may show this may be the case, but we can broaden this concept to include failure of neuromuscular reciprocal interaction. This could include failure of motor axon and muscle to respond to trophic molecules due to reduced or aberrant receptor synthesis, impaired downstream signaling or axonal transport. Axon degeneration, from distal synaptic compartments, has been described as an early event in both human disease and animal models (7, 8). These observations support the “dying back” hypothesis by which the degeneration of the NMJ and associated muscle function precede the death of motor neurons and contributes to the disease process (7–9). Moreover, the notion of non-cell autonomous degeneration in ALS involves defects not just confined to the glial cells but retained by the muscle as well. ALS has been associated with alterations of energy homeostasis induced by mitochondrial muscle breakdown (10), and by trophic factors such as insulin-like growth factor-1 (IGF-1) and glial cell-derived neurotrophic factor that are secreted by skeletal muscle, and are known to stabilize the NMJ and thereby promote motoneuron survival (11, 12). A recent study of motor axonopathy induced in mice by overexpression of an inhibitory binding protein for IGF-1, led to the suggestion that a defect in well-known neurotrophic and myotrophic effects of IGF-1 might be common to both diabetic neuropathy and ALS (13). There is no doubt that disruption of the NMJ, seen as fasciculation and motor unit enlargement is an early feature of ALS (14, 15), and associations between excessive motor activity or enlarged motor units and the development of ALS have been recognized (16). Thus muscles, similarly to glial cells, can promote a vicious cycle of energy impairment and lack of trophic factor release that interacting with other systems, when set in motion, amplify their own processes and may accelerate the development of ALS.

Whether disease primarily affects muscle or motoneurones is critical to disease management, but this progression may be an endpoint that reveals little about its origins. In this regard, the paper by Tai et al. (1) prompts us to reevaluate the role of the peripheral target in neurodegenerative diseases where attention has traditionally been focused centrally.

## Author Contributions

IJ wrote the initial draft. PL revised the initial draft and contributed further writing. Both the authors collected data from literature and revised the final manuscript.

## Conflict of Interest Statement

The authors declare that the research was conducted in the absence of any commercial or financial relationships that could be construed as a potential conflict of interest.

## References

[1] Tai H Cui L Guan Y Liu M Li X Huang Y Amyotrophic lateral sclerosis and myasthenia gravis overlap syndrome: a review of two cases and the associated literature. Front Neurol (2017) 8:218. 10.3389/fneur.2017.00218 28588549 PMC5439131

[2] Hamburger V Regression versus peripheral control of differentiation in motor hypoplasia. Am J Anat (1958) 102:365–409. 10.1002/aja.1001020303 13617221

[3] Hollyday M Hamburger V. Reduction of the naturally occurring motor neuron loss by enlargement of the periphery. J Comp Neurol (1976) 170:311–20. 10.1002/cne.901700304 993371

[4] Gould TW Oppenheim RW. Motor neuron trophic factors: therapeutic use in ALS? Brain Res Rev (2011) 67:1–39. 10.1016/j.brainresrev.2010.10.003 20971133 PMC3109102

[5] Kariya S Obis T Garone C Akay T Sera F Iwata S Requirement of enhanced Survival Motoneuron protein imposed during neuromuscular junction maturation. J Clin Invest (2014) 124:785–800. 10.1172/JCI72017 24463453 PMC3904626

[6] Tovar YRLB Ramirez-Jarquin UN Lazo-Gomez R Tapia R. Trophic factors as modulators of motor neuron physiology and survival: implications for ALS therapy. Front Cell Neurosci (2014) 8:61. 10.3389/fncel.2014.00061 24616665 PMC3937589

[7] Fischer LR Culver DG Tennant P Davis AA Wang M Castellano-Sanchez A Amyotrophic lateral sclerosis is a distal axonopathy: evidence in mice and man. Exp Neurol (2004) 185:232–40. 10.1016/j.expneurol.2003.10.004 14736504

[8] Frey D Schneider C Xu L Borg J Spooren W Caroni P. Early and selective loss of neuromuscular synapse subtypes with low sprouting competence in motoneuron diseases. J Neurosci (2000) 20:2534–42. 10729333 10.1523/JNEUROSCI.20-07-02534.2000 PMC6772256

[9] Musaró A. Understanding ALS: new therapeutic approaches. FEBS J (2013) 280:4315–22. 10.1111/febs.12087 23217177

[10] Dupuis L Gonzalez de Aguilar JL Echaniz-Laguna A Eschbach J Rene F Oudart H Muscle mitochondrial uncoupling dismantles neuromuscular junction and triggers distal degeneration of motor neurons. PLoS One (2009) 4:e5390. 10.1371/journal.pone.0005390 19404401 PMC2671839

[11] Dobrowolny G Giacinti C Pelosi L Nicoletti C Winn N Barberi L Muscle expression of a local IGF-1 isoform protects motor neurons in an ALS mouse model. J Cell Biol (2005) 168:193–9. 10.1083/jcb.200407021 15657392 PMC2171577

[12] Krakora D Mulcrone P Meyer M Lewis C Bernau K Gowing G Synergistic effects of GDNF and VEGF on lifespan and disease progression in a familial ALS rat model. Mol Ther (2013) 21:1602–10. 10.1038/mt.2013.108 23712039 PMC3734670

[13] Rauskolb S Dombert B Sendtner M. Insulin-like growth factor 1 in diabetic neuropathy and amyotrophic lateral sclerosis. Neurobiol Dis (2017) 97:103–13. 10.1016/j.nbd.2016.04.007 27142684

[14] de Carvalho M Kiernan MC Swash M. Fasciculation in amyotrophic lateral sclerosis: origin and pathophysiological relevance. J Neurol Neurosurg Psychiatry (2017). 10.1136/jnnp-2017-315574 28490504

[15] Dharmadasa T Henderson RD Talman PS Macdonell RA Mathers S Schultz DW Motor neurone disease: progress and challenges. Med J Aust (2017) 206:357–62. 10.5694/mja16.01063 28446118

[16] Gordon T Hegedus J Tam SL. Adaptive and maladaptive motor axonal sprouting in aging and motoneuron disease. Neurol Res (2004) 26:174–85. 10.1179/016164104225013806 15072637

